# MEF2 transcription factors: developmental regulators and emerging cancer genes

**DOI:** 10.18632/oncotarget.6223

**Published:** 2015-10-25

**Authors:** Julia R. Pon, Marco A. Marra

**Affiliations:** ^1^ Canada's Michael Smith Genome Sciences Centre, BC Cancer Agency, Vancouver, Canada; ^2^ Department of Medical Genetics, University of British Columbia, Vancouver, Canada

**Keywords:** MEF2, transcription factor, cancer, gene regulation, developmental biology

## Abstract

The MEF2 transcription factors have roles in muscle, cardiac, skeletal, vascular, neural, blood and immune system cell development through their effects on cell differentiation, proliferation, apoptosis, migration, shape and metabolism. Altered MEF2 activity plays a role in human diseases and has recently been implicated in the development of several cancer types. In particular, MEF2B, the most divergent and least studied protein of the MEF2 family, has a role unique from its paralogs in non-Hodgkin lymphomas. The use of genome-scale technologies has enabled comprehensive MEF2 target gene sets to be identified, contributing to our understanding of MEF2 proteins as nodes in complex regulatory networks. This review surveys the molecular interactions of MEF2 proteins and their effects on cellular and organismal phenotypes. We include a discussion of the emerging roles of MEF2 proteins as oncogenes and tumor suppressors of cancer. Throughout this article we highlight similarities and differences between the MEF2 family proteins, including a focus on functions of MEF2B.

## INTRODUCTION

The MEF2 transcription factors have a diversity of functions in a wide range of tissues and have been implicated in numerous diseases. Alterations affecting MEF2 proteins have long been known to contribute to development and neurological disorders but more recently have been implicated as drivers of cancer development. Moreover, the regulatory networks of MEF2 proteins are now being characterized in unprecedented detail. However, differences between the activities of MEF2 proteins are often not readily discernible in research reports. MEF2B, the most divergent of the MEF2 family, has received the least attention perhaps because of the difficulties of generating MEF2B specific reagents. However, MEF2B was the only MEF2 family member strongly implicated in lymphoma development, indicating that its distinct features are relevant to human disease.

Within the last ten years, functions of MEF2 proteins have been reviewed in particular cell types (e.g. neurons [1–3], muscle [4, 5] and hematopoietic cells [6]) or from the perspective of a particular discipline (e.g. developmental biology [7]). This review surveys findings from across tissue types and from molecular, cellular and organismal levels. We then discuss the roles of MEF2 protein in disease processes, with a focus on the latest implications of MEF2 proteins in cancer development. Throughout this discussion we highlight advances made using genome-scale technologies and distinguish activities of each of the MEF2 proteins. We attend in particular to how the functions of MEF2B compare with those of its paralogs. Overall, the activities of MEF2 proteins exemplify the context dependent and pleiotropic effects that transcription factors can have in normal and disease tissues.

## MEF2 FAMILY PROTEINS

The myocyte enhancer factor 2 (MEF2) family of human transcription factors consists of four proteins, MEF2A, -B, -C and -D, each of which has a homolog in other vertebrates [7]. *MEF2A* and *-C* have the most similar sequences, likely resulting from a duplication event that occurred near the origin of vertebrates [8]. In contrast, *MEF2B* appears to be the first of the *MEF2* family to have diverged from a single ancestral *MEF2* gene [8]. The commonly used model organisms *S. cerevisiae*, *C. elegans*, and *D. melanogaster* contain only one *MEF2* family gene [7].

All MEF2 proteins contain three domains: an N-terminal DNA-binding MADS domain, a central MEF2 domain and a C-terminal transactivation domain [7]. The MADS and MEF2 domains are well conserved across the MEF2 family, with 91% and 68% amino acid identity, respectively, between MEF2A and the most divergent MEF2 protein, MEF2B [7]. The transactivation domain is less well conserved, with only 6% amino acid identity between MEF2A and -B [7].

## ROLES OF MEF2 PROTEINS IN VERTEBRATE ORGANISMS

**Figure 1:**
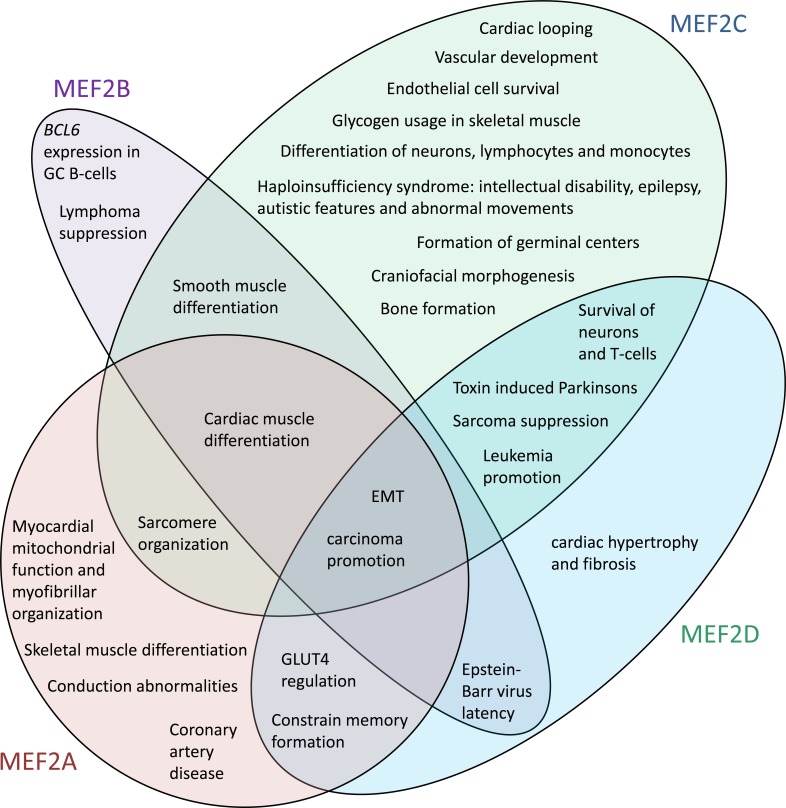
The human MEF2 proteins have distinct but overlapping sets of functions References for noted functions are provided throughout the text. A function is noted for a MEF2 protein only where it has been demonstrated for that MEF2 protein. The lack of indication that a MEF2 protein is involved in a function may be because the capacity of that MEF2 protein to regulate that function has not yet been investigated.

MEF2 family proteins play central roles in the differentiation, morphogenesis, and maintenance of several vertebrate tissue types (Figure 1). The MEF2 proteins were named myocyte enhancer factors because of their roles in muscle cell differentiation [9]. MEF2A cooperates with other factors to promote skeletal muscle differentiation [10–12] and MEF2C promotes differentiation of mouse smooth muscle cells [13]. Interestingly, MEF2B mediates de-differentiation of vascular smooth muscle cells in response to cyclic stretch [14]. Multiple MEF2 proteins are likely involved in cardiac muscle differentiation, as competitive inhibition of binding to MEF2 sites impaired cardiac muscle differentiation [15] but knockouts of individual *Mef2* genes did not [16–18]. Indeed, MEF2A, B and -C have all been implicated in cardiac muscle differentiation [19–23].

MEF2C is also required for normal neural differentiation. Mice with a *Mef2c* deletion in neural progenitor cells had less mature neurons, smaller brain sizes and severe behavioral abnormalities [24]. Conversely, expression of constitutively active MEF2C caused embryonic stem cells to differentiate into neurons [25]. Furthermore, MEF2 proteins have roles in hematopoetic cell differentiation. MEF2C promotes precursor cell commitment towards lymphoid rather than myeloid lineages and promotes development towards monocyte rather than granulocyte fates [6]. In B-cells, MEF2C activity is necessary for germinal centre formation [26, 27] and MEF2B and MEF2D are involved in maintaining Epstein-Barr virus latency [28, 29]. Interestingly, the *Drosophila* MEF2 protein coordinates immune functions with metabolic activities. Infection triggers the de-phosphorylation of MEF2 proteins in *Drosophila* fat pads, causing MEF2 proteins to switch from promoting the expression of enzymes involved in anabolic metabolism to instead promoting the expression of anti-microbial peptides [30]. Recruitment of MEF2 to anti-microbial peptide genes occurs through the association of the unphosphorylated form of MEF2 with TATA binding proteins. A role for human MEF2A and MEF2D in regulating expression of the glucose transporter gene *GLUT4* has been confirmed [31] and glycogen was aberrantly accumulated in the muscle of *Mef2c* knockout mice [32].

Other activities of MEF2A, -C and -D relate to the regulation of cytoskeletal structures. For instance, deletion of *Mef2c* in skeletal muscle cells resulted in sarcomere disorganization [33]. Neuronal cytoskeletal structures are regulated in part by MEF2A and MEF2D. Indeed, MEF2A and MEF2D constrain memory formation through their suppression of dendritic spine and excitatory synapse formation [34–36]. MEF2 proteins also contribute to the formation of large-scale tissue structures. For instance, *Mef2c* null mice have cardiac looping defects [16] and mice heterozygous for *Mef2c* deletion had decreased ossification and impaired chondrocyte hypertrophy in the sternum [18]. Neural crest-specific deletion of *Mef2c* produced defects in craniofacial morphogenesis [37]. The formation of craniofacial structures requires neural crest precursor cells to undergo epithelial-mesenchymal transition (EMT) and migrate to appropriate locations [38]. As MEF2A, -C and -D promoted EMT of hepatocellular carcinoma cells [39], they may also promote EMT of the neural crest cells.

Finally, MEF2A, -C and -D have roles in regulating apoptosis. MEF2C activity in endothelial cells [40] and MEF2A, -C and -D activity in developing neurons [41–43] inhibits apoptosis downstream of mitogen-activated protein kinase signaling. Interestingly, in mature neurons exposed to stress the anti-apoptotic effects of MEF2 proteins can be overcome by activation of caspase-mediated cleavage of the MEF2A, -C and -D transactivation domains [43, 44]. Some of the remaining fragments contain dimerization domains that can inhibit the activity of intact MEF2 proteins in a dominant negative fashion [44]. In contrast, in T-cells MEF2C and MEF2D actively promote apoptosis downstream of T-cell receptor signaling by promoting the expression of *Nur77* [45–47]. Although MEF2 proteins also promote *Nur77* expression in neurons, functions of NUR77 in neurons relate to the inhibition of synaptic structure formation rather than the promotion of apoptosis [48, 49].

Knockout mouse models have also provided insight into the potential redundancy between MEF2 proteins. *Mef2a* [17] and *Mef2c* [50] null mice exhibited neonatal and embryonic lethality, respectively. Thus, MEF2A and MEF2C each perform some functions that cannot be adequately performed by other MEF2 proteins. Specifically, *Mef2a* null mice exhibited myocardial mitochondrial defects [17] whereas *Mef2c* null mice failed to undergo normal cardiac morphogenesis [50] and had severe vascular abnormalities[13]. In contrast, mice null for either *Mef2b* [16] or *Mef2d* [51] were viable and had no obvious abnormalities. Thus, either MEF2B and MEF2D do not contribute to development or other MEF2 proteins are able to compensate for the loss of MEF2B or MEF2D activity during development. However, adult *Mef2d* knockout mice showed a reduced response to stress signals that would normally trigger cardiac hypertrophy and fibrosis [51], indicating MEF2D's activities are not entirely redundant.

Interestingly, the expression pattern of *Mef2b* mRNA indicates that MEF2B, like its paralogs, may contribute to development and maintenance of a variety of tissue types. Similar to other *Mef2* genes, *Mef2b* mRNA transcripts have been detected in developing cardiac muscle cells, skeletal muscle cells, neurons, neural crest cells, whisker follicle cells and chondrocytes of mouse embryos [52]. Rodent *Mef2b* mRNA expression has also been reported in the proliferating smooth muscle cells of injured arteries [53] and in the brain's cortex, olfactory blub and amygdala [1]. Human *MEF2B* mRNA expression has been detected in B-cell and T-cell lymphomas [54]. Further investigation indicated that *MEF2B* mRNA is expressed in germinal centre (GC) B-cells but not in naïve B-cells or in B-cells that have differentiated into plasma cells [54, 55]. Of the mouse MEF2 genes, *Mef2c* has the most similar expression pattern to MEF2B and may thus have the most similar cellular functions [52]. However, the cell types containing appreciable levels of MEF2B protein may be a subset of those expressing *MEF2B* mRNA, as the translation of other *MEF2* mRNAs is known to be suppressed in many tissue types [56]. MEF2B protein has been detected in GC B-cells, fibroblasts, myoblasts, myotubes and vascular smooth muscle cells [52, 53, 55, 57].

## GENOME-WIDE TARGET GENE IDENTIFICATION

Attempts to better understand the role of MEF2 proteins have included attempts to identify MEF2 target genes throughout the genome. The target genes identified for different MEF2 proteins in different cell types tended to be enriched for different functional annotation groups. For instance, a study of MEF2A DNA-binding sites in cardiomyocytes reported that candidate direct target genes were enriched for functions related to heart and muscle development and cytoskeleton organization [20]. In contrast, a study of MEF2A and -D in hippocampal neurons identified target genes that tended to have functions at neural synapses and expression only in central nervous system cells [58]. Effects of a constitutively active MEF2 protein on global gene expression patterns in human neural progenitor cells were also recently assessed [59]. A study of the role of MEF2C in bone formation found that genes associated with MEF2C binding sites were enriched for genes that regulate bone turnover [60]. In HEK293 cells, MEF2B target genes were enriched for regulators of cell migration and genes involved in epithelial-mesenchymal transition. Target genes of MEF2B also included the cancer genes *MYC*, *TGFB1*, *CARD11*, *RHOB* and *NDRG1.* Other ChIP-seq datasets available include those produced by the ENCODE consortium for MEF2A and MEF2C in GM12878 lymphoblastoid cells and for MEF2A in K562 myelogenous leukemia cells [61].

One study has directly compared target gene sets of each of the MEF2 proteins in mouse myoblasts [12]. In that study, genes whose expression levels were altered by reducing levels of a MEF2 protein were considered candidate target genes of that MEF2 protein. The numbers of candidate target genes ranged from 110 for MEF2D to 4,020 for MEF2A. Of the candidate target genes for one MEF2 protein, 10% to 81% were not candidate target genes of any other MEF2 protein. These differences between target gene sets are consistent with evidence that each MEF2 protein has some cellular functions distinct from those of the other MEF2 proteins. The 21 target genes shared by all four MEF2 proteins were enriched for regulation by calpain proteases, neural NOS signaling, integrin signaling, amyloid processing and FAK signaling pathways.

The notion that MEF2 proteins have target genes unique from those of their paralogs is also supported by earlier studies identifying differences between the target genes of MEF2B and those of other MEF2 proteins. MEF2B was the only MEF2 protein to bind a region required for maintaining *SMHC* expression [62] and MEF2B overexpression but not MEF2D overexpression increased *BZLF1* transcription [29]. Similarly, MEF2B overexpression but not MEF2A or -C overexpression increased *SOST* expression downstream of the *ECR5* enhancer region [63]. Conversely, MEF2B may not regulate some genes that are direct targets of other MEF2 proteins: MEF2B was the only MEF2 protein that did not bind regulatory sequences near *NUR77* [64] or the immunoglobulin J chain gene [65].

## FUNCTIONS OF THE MADS AND MEF2 DOMAINS IN MEF2 PROTEINS

The MADS box is a region of 56 amino acids highly conserved across the MADS family proteins [66], whereas the MEF2 domain is a 29 amino acid region unique to MEF2 family proteins [67]. Both the MADS and MEF2 domains are required for DNA-binding [68]. Notably, MEF2 proteins bind DNA as dimers and the MADS and MEF2 domains are essential for dimerization [68]. Complexes thought to represent MEF2A-MEF2D heterodimers, MEF2C-MEF2D heterodimers and MEF2D homodimers have been identified in HEK293 cells [69]. Although the binding site motifs generated for MEF2A and C in lymphoblastoid cells [61] are nearly identical to that generated for MEF2B in HEK293A cells [70], DNA binding affinity may differ between MEF2 proteins. In particular, MEF2B is the only MEF2 protein to contain glutamine (Q) rather than glutamic acid (E) at residue 14. Q14E mutation of MEF2B increased DNA binding by approximately two-fold [52], consistent with the notion that MEF2B may have slightly reduced affinity for DNA binding sites when compared to other MEF2 proteins.

Dissociation constants for DNA-binding have been determined for MEF2A [71] and MEF2C [72, 73]. However, these studies used MEF2 proteins expressed in bacteria, where the MEF2 proteins escaped post-translational modifications that modulate their DNA binding affinity. For instance, casein kinase II phosphorylates S59 of MEF2C, a modification that enhances MEF2C DNA binding by five-fold [74]. Similarly, acetylation of K4 increases MEF2C DNA binding [75]. S59 and K4 are conserved in MEF2A, -B and -D, though their post-translational modification has only been investigated in MEF2C. Cell type specific factors may also influence DNA binding specificity. Indeed, in neuronal cells compared to muscle cells, MEF2A showed additional constraints for DNA-binding based on the sequences flanking MEF2 motifs [76].

The MEF2 domain is also involved in interactions with co-activators and co-repressors. Co-repressors that are thought to associate with the MEF2 domains of all MEF2 family proteins include the class IIa histone deacetylases HDAC4, -5, -7 and -9 [77–80]. Although class IIa HDACs have minimal deacetylase activity [81, 82], they can mediate transcriptional repression by recruiting other co-repressors such as HP-1, CtBP and class I HDACs [82–84]. Another co-repressor interacting directly with the MEF2 domain is CABIN1 [46, 85]. CABIN1 also interacts with class I HDACs [46] and can interact with the H3K9 methyltransferase SUV39H1 [86]. Co-activators binding the MEF2 domains of MEF2A [87], -C [88] and -D [46, 89] include the histone acetyltransferases CREBBP and p300, which are structural and functional homologs [90].

Given these interactions with histone modifying enzymes, MEF2 proteins may alter expression of their target genes by promoting changes in histone modification. Alternatively, HDACs and p300 may modulate MEF2 target gene expression by altering acetylation states of MEF2 proteins themselves. Deacetylation of MEF2D by HDAC4 allows MEF2D to be sumoylated [91]. Sumoylation inhibits the capacity of MEF2 proteins to activate transcription [91]. Conversely, p300 can acetylate MEF2C, promoting MEF2C's transcriptional activity [75, 92]. p300 may also play a structural role linking MEF2 proteins to other transcription factors and transcriptional machinery, as p300 can interact with basal transcription factors and RNA polymerase II [93].

Because their binding sites on MEF2 proteins overlap, CABIN1, class IIa HDACs, CREBBP and p300 may compete to bind MEF2 proteins [46]. Indeed, decreased interaction of CABIN1 with MEF2D correlated with an increase in the interaction of MEF2D with p300 [46]. Association of HDACs and CABIN1 with MEF2D is inhibited by increased intracellular calcium levels [46]. Specifically, high calcium levels promote nuclear export of CABIN1 and class II HDACs [94–96] and cause CABIN1 and class II HDACs to be sequestered into complexes with calcium-calmodulin [46]. Regulation of these and other co-repressors by calcium may explain why the expression of some MEF2 target genes is calcium sensitive (reviewed in McKinsey *et al*., 2002). For instance, *Nur77* expression in T-cells is dependent on the presence of MEF2 binding sites and is induced by calcium signaling [97]. Similarly, treatment with a calcium ionophore increased MEF2-dependent luciferase expression in T-cells [47].

MEF2-dependent gene expression may also be regulated by calcium signaling downstream of B-cell receptor (BCR) activation in B-cells. Consistent with this notion, most gene expression differences between mice with B-cell specific *Mef2c* deletions and control mice were evident only when BCR signaling was activated [27]. Specifically, activation of BCR signaling tended to increase MEF2C target gene expression in control B-cells but not in MEF2C deficient B-cells. However, BCR signaling involves multiple signal transduction pathways, including activation of p38 via protein kinase C (PKC) [98]. p38 can phosphorylate all MEF2 proteins, including MEF2B [69]. In muscle cells, phosphorylation by p38 promotes association of MEF2C and MEF2D with the histone methyltransferase KMT2D (also known as MLL2 and MLL4) [99]. p38 and calcium signaling may thus contribute synergistically to regulation of MEF2 target gene expression. This notion is supported by evidence that the treatment of cells with both the calcium ionophore ionomycin and the PKC activator PMA produced greater MEF2-dependent luciferase expression than treatment with either agent alone [47].

Other transcription factors can also cooperate with MEF2 proteins through interaction with the MADS or MEF2 domains. A well-studied example is the interaction of MEF2 proteins with Myogenic Regulatory Factors (MRFs). MEF2A, -C and -D only induced muscle gene expression in transfected fibroblasts when a MRF protein was co-expressed [11]. Once associated with MRFs, MEF2 proteins are thought to promote interactions between MEF2-MRF complexes and transcriptional machinery [100]. Interestingly, even though interaction with MRFs occurs through the MADS box of MEF2 proteins, MEF2 DNA-binding activity is not required for induction of a muscle gene expression program [11]. Similarly, the MADS and MEF2 domains of MEF2A, -C and -D can interact with GATA transcription factors to synergistically activate cardiac-specific gene expression, without requiring MEF2 DNA-binding capacity [101]. Thus, MEF2 proteins may affect expression of genes without MEF2 binding sites, through interactions with other transcription factors.

## FUNCTIONS OF THE TRANSACTIVATION DOMAINS OF MEF2 PROTEINS

The MADS and MEF2 domains of MEF2 proteins are sufficient to recruit certain coregulators (discussed above), but are not sufficient to strongly activate the expression of all target genes. For instance, MEF2B and MEF2C proteins containing the MADS and MEF2 domains but lacking most of their transactivation domain had eliminated and reduced capacities, respectively, to activate expression of a MEF2-dependent reporter gene [52, 68]. Interestingly, when the MADS and MEF2 domains of MEF2C were replaced with a GAL4 DNA binding domain, the resulting fusion protein could activate the expression of a reporter gene whose promoter contained a GAL4 binding site [68]. Thus, the coregulators that interact with the MADS and MEF2 domains are not essential for MEF2C to activate the expression of some target genes. Rather, coregulators recruited by the MADS and MEF2 domains may modulate the degree of target gene activation.

The mechanisms by which MEF2 transactivation domains activate transcription remain unclear. One possible mechanism is through interaction with the positive transcription elongation factor b (P-TEFb), which hyperphosphorylates the C-terminal region of RNA polymerase II to promote transcription [102]. P-TEFb was co-immunoprecipitated with MEF2A, -C and -D [103] and overexpression of P-TEFb increased MEF2-dependent transcription. Furthermore, P-TEFb was recruited to MEF2 binding sites when MEF2-dependent transcription was activated. However, it remains unknown which domains of MEF2 proteins are required for interaction with P-TEFb.

An additional function of MEF2 transactivation domains is to integrate regulatory signals. Numerous sites of post-translational modification have been identified in the transactivation domains, including phosphorylation sites for p38 [69, 104] (discussed above), BMK1 [105] and PKA [55, 106]. BMK1 phosphorylates and activates MEF2A, -C and -D but not MEF2B [105]. In contrast, both MEF2B and MEF2D are phosphorylated by PKA [55, 106]. Phosphorylation at some sites in the MEF2B, -C and -D transactivation domains promotes sumoylation at nearby residues. and decreases MEF2-dependent reporter gene expression [55, 107, 108]. However, only *MEF2C* contains a splice acceptor site that allows its sumoylation site to be spliced out. MEF2C isoforms lacking the sumoylation site thus escape repression [109]. MEF2 protein phosphorylation can also promote ubiquitination. Specifically, phosphorylation by CDK4/cyclin D1 at S98 and S110 allows MEF2D to interact with the E3 ligase SKP2, which mediates the ubiquitination and degradation of MEF2C and -D [110]. Degradation of MEF2C and -D via this mechanism de-represses progression into S phase [110].

Alternative splicing may also alter the transactivation domains of MEF2 proteins in other ways. For instance, MEF2A, -C and -D transcripts in striated muscle and neural tissue may include a β exon that increases the capacity of the encoded protein to activate transcription [111]. However, the mechanism by which exon β inclusion increases activity remains unclear. More clearly understood are the effects of the alternative third exons, α1 and α2, of MEF2A, -C and -D. These exons encode an amino acid sequence immediately C-terminal to the MEF2 domain. MEF2Cα2 is predominantly expressed in skeletal muscle [112] and promotes muscle-specific gene expression and myogenic differentiation to a greater extent than MEF2Cα1, perhaps because of its decreased association with the corepressor HDAC5 [113].

In contrast to the many isoforms of MEF2A, -C and -D, only two isoforms of MEF2B have been reported: isoforms A and B [55]. Isoform A MEF2B includes all exons, whereas isoform B excludes exon 8. The exclusion of exon 8 results in a frameshift that alters all amino acids C-terminal to those encoded by exon 7. Consequently, 40% of the amino acids in the transactivation domain of isoform A are altered in isoform B, reducing the capacity of isoform B MEF2B to activate transcription [70].

## ROLES OF MEF2 FAMILY PROTEINS IN HUMAN DISEASE

Of the MEF2 proteins, MEF2C has been associated with the widest range of disorders. For instance, increased MEF2C abundance has been associated with congenital heart defects [114] and decreased MEF2C abundance produces MEF2C haploinsufficiency syndrome. This syndrome is characterized by intellectual disability, epilepsy, autistic features and abnormal movements [115, 116]. Abnormal movement is also a characteristic of Parkinson's disease, the toxin-induced form of which has been associated with decreased MEF2C and -D activity [117, 118]. Alterations affecting MEF2 target genes have been implicated in other neurological disorders including autism spectrum disorders [119], Alzheimer's disease [120] and Angelman syndrome [121]. Thus, alterations affecting MEF2 proteins may also impact these diseases. In contrast to the neurological symptoms associated with decreased MEF2C and -D, decreased activity of MEF2A has been associated with an autosomal dominant form of coronary artery disease [122]. The only non-cancer disease or disorder in which MEF2B alterations may be implicated is intellectual disability, based on the weak evidence that *MEF2B* was co-deleted with 10 or 75 other genes in two patients with intellectual disability [123].

## ONCOGENIC ACTIVITY OF MEF2 FAMILY GENES

Roles of MEF2 proteins in cancer development have only recently been investigated (Table 1). MEF2 family genes are most well characterized as oncogenes of hematological cancers. For instance, increased *MEF2C* expression is characteristic of immature T-cell acute lymphoblastic leukemia [124]. B-cell acute lymphoblastic leukemias also exhibit recurrent rearrangements producing DAZAP1/MEF2D fusion proteins that have oncogenic activity [125]. In mouse models of myeloid leukemia, *Mef2c* was able to act as a co-operating oncogene in combination with either *Irf8* deficiency [126] or *Sox4* activation [127], though it was insufficient to independently drive leukemia development. Myeloid leukemias initiated through MLL-AF9 displayed increased *Mef2c* expression and required elevated MEF2C levels to maintain a high capacity for colony formation [128]. As MEF2C is normally expressed in myeloid progenitor cells, it may be involved in conferring stem-cell like properties [6]. MEF2C also promoted the migration and invasion of leukemic cells [126]. In contrast to MEF2C and MEF2D, MEF2A and MEF2B have not been implicated in leukemia development.

**Table 1:** Effects of MEF2 alterations in cancers

Cancer type	MEF2 protein involved	Role of MEF2 protein	Alteration of MEF2 protein	Effects of MEF2 alteration	References
Immature T-cell acute lymphoblastic leukemia	MEF2C	Oncogene	Increased expression due to rearrangements or alterations affecting interacting proteins.	Inhibition of differentiation.	[124]
B-cell acute lymphoblastic leukemia	MEF2D	Oncogene	Fusion with DAZAPI.	Promotion of colony formation and proliferation in low serum conditions. Inhibition of apoptosis.	[125]
Myeloid leukemia	MEF2C	Oncogene	Expression activated by retroviral insertion in mouse model. Increased expression in patient samples with *MLL* rearrangements.	Promotion of colony formation, migration, invasion and stem-cell-like properties.	[126–128]
Hepatocellular carcinoma	primarily MEF2C and MEF2D	Oncogene	Increased expression.	Epithelial-mesenchymal transition and invasiveness. Variable effects on proliferation.	[39, 129, 130, 133]
Pancreatic ductal adenocarcinoma	MEF2C	Oncogene	Increased expression resulting from decreased YY1 expression.	Promotion of *MMP10* expression and invasiveness.	[136]
Lipo- and leiomyosarcoma	MEF2C, MEF2D	Tumor suppressor	Decreased MEF2 activity and abundance resulting from increased HDAC4 and PI3K/AKT activity.	Promotion of cell proliferation and anchorage independent growth.	[141]
Rhabdomyosarcoma	MEF2C, MEF2D	Tumor suppressor	Loss of MEF2D expression. Increased ratio of MEF2Cα1 (less active isoform) compared to MEF2Cα2.	Inhibition of differentiation. Promotion of cell proliferation, anchorage independent growth and cell migration.	[113, 142]
Diffuse large B-cell lymphoma and follicular lymphoma	MEF2B, very rarely MEF2C	Tumor suppressor	Nonsynonymous mutations in the MADS and MEF2 domains with hotspots at K4, Y69 and D83. Primarily nonsense, frameshift and stop codon read-through mutations in the transactivation domains.	May de-repress chemotaxis. May promote *MYC* and *TGFB1* expression.	[55, 70, 143–146]
Mantle cell lymphoma	MEF2B	Unknown	Primarily K23R mutations.	Unknown.	[147, 148]

Roles for MEF2 proteins in solid tumor development have also been proposed. MEF2A and -C mRNA and protein abundance tended to be greater in hepatocellular carcinoma (HCC) cells than normal liver cells [129] and MEF2D expression in HCC patient samples was associated with poor prognosis [130]. Providing further evidence that MEF2 activity may drive HCC development, 40 out of 193 HCC cases (21%) had an amplification of a MEF2 gene [131, 132]. Although MEF2D was the most frequently amplified (29/193), all three other MEF2 family genes were amplified in at least one case.

MEF2A, -C and -D promoted HCC cell invasiveness by promoting epithelial-mesenchymal transition (EMT) [39]. Interestingly, TGFβ1 is both an upstream activator of MEF2 activity and is encoded by a MEF2 target gene [39]. Thus, a positive feedback loop may help maintain invasiveness. A separate study found that nuclear MEF2C promotes VEGF-mediated HCC cell invasion and angiogenesis, whereas cytoplasmic MEF2C sequesters β-catenin in the cytoplasm, reducing the capacity of β-catenin to promote cell proliferation [133]. Thus, MEF2C, like TGFβ cytokines [134], may act as a “double-edged sword” in HCC through its promotion of cell invasion and inhibition of cell proliferation [133]. Curiously, MEF2D had an effect on proliferation opposite that of MEF2C. MEF2D overexpression increased HCC cell proliferation and MEF2D-positive HCC cells had greater proliferation rates than MEF2D-negative HCC cells [130].

**Figure 2:**
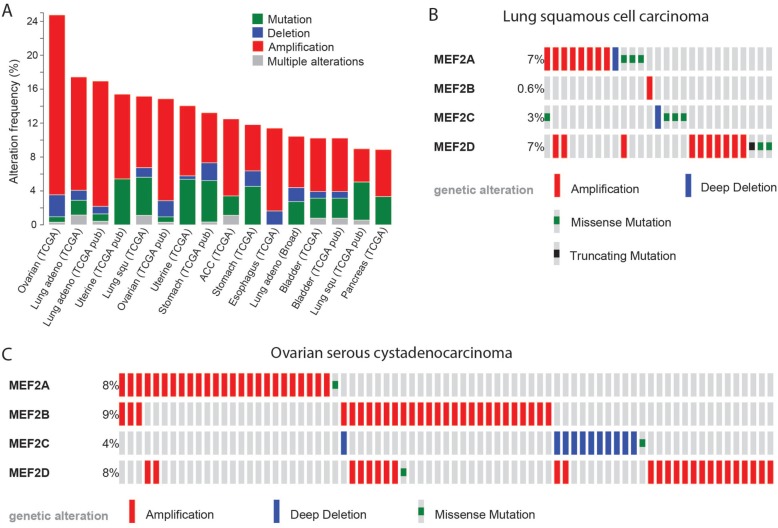
MEF2 proteins may act as oncogenes in several types of carcinoma **A.** Alterations affecting *MEF2* genes are present in up to 24% of samples of certain cancer types. Shown are data for cancer types in which *MEF2* genes were altered in over 8% of cases. In the shown cancer types, *MEF2* amplifications were more common than deletions, consistent with the notion that *MEF2* genes may act as oncogenes. **B.** Some *MEF2* genes are more frequently affected than others in certain cancer types. The plot shows how in lung squamous cell carcinoma alterations more commonly affect *MEF2A* and *-D* than *MEF2B* and *-C*. **C.** In certain cancer types, some *MEF2* genes tend to be affected by different types of alterations than other *MEF2* genes. The plot shows how in ovarian serous cystadenocarcinoma the copy number alterations affecting *MEF2C* are deletions, whereas those affecting other *MEF2* genes are amplifications. Data and plots were obtained using cBioportal [131, 132].

As some degree of EMT is necessary for the invasion and dissemination of carcinoma cells [135], *MEF2* genes may also have oncogenic roles in the development of other types of carcinoma. Indeed, particularly poor prognosis in pancreatic ductal adenocarcinoma was associated with decreased expression of YY1, a suppressor of *MEF2C* expression [136]. Decreased YY1 expression increased the invasiveness of pancreatic adrenocarcinoma cells through MEF2C-mediated activation of MMP10 expression [136, 137]. Though not functionally characterized, roles for MEF2 proteins in other carcinomas may be predicted from the recurrence of alterations affecting *MEF2* genes. According to the cBioPortal database, 6 to 21% of ovarian serous cystadenocarcinomas, lung squamous cell and adenocarcinomas, uterine endometriod carcinomas, stomach adenocarcinomas, adrenocortical carcinomas, esophageal carcinomas, bladder urothelial carcinomas and pancreatic adenocarcinomas contained an amplification of a *MEF2* gene, whereas instead of but 0 to 2.6% of these cancers contained a *MEF2* gene deletion [131, 132, 138–140] (Figure 2A). *MEF2* genes thus may have the potential to act as oncogenes in these carcinomas. The cBioPortal data also illustrates how in some cancer types different MEF2 family genes have alterations at the different frequencies (Figure 2B) or tend to be affected by different types of alterations (Figure 2C). Even the most divergent family member, *MEF2B*, had amplifications in 3 to 9% of ovarian serous cystadenocarcinomas, adrenocortical carcinomas, and esophageal carcinomas [131, 132], indicating that it too may have oncogenic activity. Indeed, MEF2B promoted a mesenchymal gene expression signature and increased cell migration when overexpressed in HEK293 cells, consistent with the notion that it too promotes EMT [70].

## MEF2 FAMILY PROTEINS AS TUMOR SUPPRESSORS

Tumor suppressor activities of MEF2 proteins have also been identified. For instance, MEF2 target gene expression tended to be decreased in lipo- and leiomyosarcomas compared to normal tissue [141]. This repression correlated with decreased MEF2C abundance and increased activity of negative regulators of MEF2 activity (i.e. HDAC4 and PI3K/AKT signaling). Inhibition of PI3K/AKT signaling and MEF2-HDAC interactions synergistically restored expression of MEF2 target genes and decreased leiomyosarcoma cell line proliferation. MEF2C and MEF2D may also act as tumor suppressors in rhabdomyosarcoma. In rhabdomyosarcoma cells compared to normal myoblasts, *MEF2D* expression tended to be lost and a less active isoform of MEF2C tended to be expressed [113, 142]. The expression of exogenous *MEF2D* in rhabdomyosarcoma cell lines promoted differentiation and inhibited cell proliferation, anchorage independent-growth and cell migration [142].

Roles of MEF2 proteins in non-Hodgkin lymphomas have also been identified. *MEF2B* is the target of heterozygous somatic non-synonymous and indel mutations in 8 to 18% of diffuse large B-cell lymphoma (DLBCL) [143–146], 13% of follicular lymphoma (FL) [143] and 3 to 7% of mantle cell lymphoma (MCL) [147,148]. Other MEF2 proteins were much less commonly affected in lymphoma [143, 144], indicating that MEF2B has a role unique from its paralogs in the B-cells from which these lymphomas arise. Mutations in the MADS and MEF2 domains represented 79% of *MEF2B* mutations in DLBCL [143–146], 75% of *MEF2B* mutations in FL [143] and 93% of *MEF2B* mutations in MCL [147, 148]. Mutation hotspots in DLBCL and FL were present at K4 (7% of mutations), Y69 (8% of mutations) and D83 (33% of mutations) [143–146]. In contrast, *MEF2B* mutations in MCL were predominantly K23R mutations (10 out of 14 mutations) [147, 148]. Why the mutation spectrum differs between MCL and other lymphomas remains to be determined. The recurrence of *MEF2B* mutations at particular residues is consistent with the notion that *MEF2B* mutations have either gain-of-function or dominant negative effects on MEF2B activity.

Although an early study found that some *MEF2B* mutations increased the expression of a *BCL6* reporter gene in HEK293 cells by disrupting interactions with the co-repressor CABIN1 [55], a subsequent study found that *MEF2B* mutations tended to decrease endogenous MEF2B target gene activation in both HEK293 and DLBCL cells [70]. For the K4E and D83V mutations, decreased transcriptional activity resulted in part from decreased DNA binding. The notion that *MEF2B* mutations decrease direct target gene activation is also consistent with the identification of two homozygous MEF2B deletions but no MEF2B amplifications in DLBCL [131, 132]. Moreover, the types of mutations common in the transactivation domain (i.e. nonsense, frameshift, stop codon read-through and splice site mutations) are typically inactivating [143]. Such inactivating mutations may not occur in the MADS and MEF2 domains as the capacity of the mutant proteins to dimerize must be preserved in order for them to have dominant negative effects. Although some nonsense mutations in the transactivation domain can remove sites for inhibitory post-translational modifications [55], they may also remove regions necessary for the transcriptional activation of key target genes. Indeed, deletion of the C-terminal third of the MEF2B transactivation domain decreased activation of a *MCK* reporter gene in mouse embryonic fibroblasts [52]. Not only do some mutations disrupt over 90% of the transactivation domain [143], others result in constitutive production of protein almost identical to the less transcriptionally active isoform, isoform B [55].

Preliminary data indicates that loss of MEF2B activity de-represses DLBCL cell chemotaxis, indicating that the mutations may contribute to DLBCL and FL development by allowing migration outside of germinal centres [70]. *MEF2B* mutations also increased expression of the MYC oncogene and decreased expression of the TGFB1 tumor suppressor in HEK293 cells [70]. Such expression changes in DLBCL would be expected to promote lymphoma development [149–151].

## CONCLUSIONS

Transcriptional regulation by MEF2 family members is modulated by a diversity of upstream regulators, splice isoforms, post-translational modifications, and coregulatory proteins. MEF2 proteins can thus perform diverse and cell-type-specific functions. Mapping MEF2 regulatory networks in the multitude of contexts in which they act is an ongoing endeavor. Differences in the roles of each MEF2 paralog highlight the importance of developing paralog-specific reagents and making the particular paralogs studied readily ascertainable in research reports.

Improving our understanding the pathways through which each MEF2 protein acts may prove relevant to diseases of cardiovascular, neural, musculoskeletal, blood and immune system cells. Indeed, the role of MEF2 proteins in common diseases such as autism spectrum disorders, Alzheimer's disease and numerous types of cancer remains to be fully elucidated. The involvement of MEF2 proteins in both cancers and developmental disorders underscores an emerging theme that cancer genes tend to have roles in development. Communication and collaboration between biochemists, cancer biologists, geneticists and developmental biologists will thus be essential for developing of a complete picture of the roles of *MEF2* genes and other cancer genes.
